# The COVID-19 pandemic, emergency aid and social work in Brazil

**DOI:** 10.1177/1473325020981753

**Published:** 2021-03

**Authors:** Maria Lúcia T Garcia, Aline F Pandolfi, Fabiola X Leal, Aline F Stocco, Arelys Esquenazi Borrego, Rodrigo ES Borges, Edineia F dos A Oliveira, Aline EM Lang, Cenira Oliveira Andrade, Silvia N Salazar, Leila MT Menandro, Gary Spolander

**Affiliations:** Department of Social Work and of the Graduate Programme in Social Policy, Federal University of Espírito Santo, Espírito Santo, Brazil; Department of Social Work, Federal University of Espírito Santo, Espírito Santo, Brazil; Department of Social Work and of the Graduate Programme in Social Policy, Federal University of Espírito Santo, Espírito Santo, Brazil; Federal University of the Vales do Jequitinhonha and Mucuri, Teofilo Otoni, Brazil; Federal University of Espírito Santo, Espírito Santo, Brazil; Federal University of Espírito Santo, Espírito Santo, Brazil; Universidad Complutense de Madrid, Madrid, Spain; Federal University of Espírito Santo, Espírito Santo, Brazil; Federal University of Espírito Santo, Espírito Santo, Brazil; Department of Social Work, Federal University of Espírito Santo, Espírito Santo, Brazil; Department of Social Work, Federal University of Espírito Santo, Espírito Santo, Brazil; Federal University of Espírito Santo, Espírito Santo, Brazil; Faculty of Medicine, University of Keele, Keele, UK

**Keywords:** Social work practice, poverty, emergency social work, emergency aid, pandemic, covid-19

## Abstract

This essay reflects on the implementation of federal government emergency aid in Brazil in the context of the COVID-19 pandemic, highlighting elements from the work of Social Workers in the context of growing demand for the supply of material provisions. Economic and social conditions in Brazil have particularities that impact the operationalisation of this benefit, which is aimed at the poor, that add complexity and impose limits. When considering the structural limits set, this context imposes challenges on the work of Social Workers. The need to reconnect and enhance the struggle for social rights is emphasised through the different strategies of the working class.

## Introduction

This essay reflects on the implementation of Federal Government Emergency Aid in Brazil in the context of the COVID-19 pandemic, highlighting elements from the work of Social Workers in the context of growing demand for the supply of material provisions. The paper is structured in two sections: a characterisation of the country's economic and social conditions followed by a reflection on the operationalisation of Emergency Aid and the work of Social Workers.

The first case of COVID-19 in Brazil was identified on February 24th, 2020. Since then, several measures have been adopted to confront this public health emergency, such as horizontal social distancing. Some regulations have been published, such as Law No. 13,979 (Brasil, 2020c) and the Legislative Decree declaring COVID-19 as a “public calamity” (Brasil, 2020c). As for economic and social measures, we highlight Emergency Financial Aid for workers with some conditions.^1^

The current health crisis in Brazil,^2^ resulting from the COVID-19 pandemic, has itself been inserted into a broader and more complex context of aggravated economic crisis. “Decades of neoliberal policies have led to the weakening and/or dismantling of social policies, especially in the field of health” (Marques, 2020). In other words, the socio-economic crisis that coronavirus has intensified, cannot be dissociated from the cumulative effects of the application of neoliberal policies, along with the impact of the 2007/2008 financial crisis, which have yet to be overcome. It is thus part of a structural capitalist crisis (Mészáros, 2009) which has affected the country since 2015. The combined effects of the crisis and its multiple concrete manifestations - including the current health crisis - have aggravated the precarious living conditions of Brazilian workers, particularly those most impoverished, under-employed and unemployed.

In this context, there are an increased number of families, without guaranteed income, who do not meet the eligibility criteria set by the Registration System (CadÚNICO) of the Brazilian Social Assistance Policy (Brasil, 2007).^3^ The per capita income eligibility of this assistance system excludes families not considered to be in extreme poverty,^4^ although they have unmet urgent material needs in accordance with the Brazilian Federal Constitution (Brasil, 1988).^5^ Consequently, it is necessary to understand the measures imposed by the federal government in the context of the pandemic, linked with the socioeconomic context of Brazilian families and the overall limits set for their implementation.

## The pandemic and the Brazilian socio-economic context

Brazil has faced difficulties, since 2015 in increasing its GDP which had fallen by 3.55% in 2015 and 3.31% in 2016, and with an average economic growth rate of 1.09% per annum for the period 2017 to 2019 (IBGE, 2019). As a result, the unemployment rate, which was 6.8% in early 2015, reached 13.7% in the first quarter of 2017 (IBGE, 2020a). In February 2020 unemployment had reduced to 11.6%, but this still equates to 12.3 million unemployed workers (IBGE, 2020a).

Among the 93.7 million people employed in February 2020, more than 36 million were either self-employed or had no formal employment contract (IBGE, 2020a). Among workers with formal employment contracts, approximately 143,000 were hired on an intermittent basis throughout 2019.^6^ Law N^o^. 13,467, of 2017, made several aspects of the country's labour legislation more flexible, such as working hours, remuneration and contract termination (Brasil, 2017), resulting in many workers having no guarantee of maintaining their income and employment. This worsened with the arrival of the economic crisis caused by the pandemic, and these workers have become eligible for the social programmes that form part of the Social Assistance Policy. In Brazil, this policy is delivered through a Unified Social Assistance System (SUAS) that consists of a set of actions, services and social assistance benefits (Brasil, 1993).

In this assistance system, the main cash transfer programme is the Bolsa Família Programme (BFP) (Silva and Lima, 2010). The limitations of this Programme, especially in relation to its Government budget financial allocation, mean that it is unable to respond to impacts arising from the pandemic. This programme has also been criticised and threatened under the country's current fiscal austerity policies.^7^ In 2019, funding was withdrawn from almost 600,000 families out of the 13 million families who were receiving support, and who had a per capita monthly income of up to R$178.00 (US$32.96) (Ministério da Cidadania, 2020d). This points to an *invisible* demand for access to social rights, this large number of people might ordinarily seek support under emergency provisions were ineligible for support and thus invisible in terms the social security system.

This invisibility highlights the limits of programmes such as the BFP, which is focused on families in extreme poverty. This is clear when we look at the average benefit amount paid (R$191.86 (US$35.53)), when the current minimum wage in Brazil is R$1,045.00 (around US$215). This situation is further aggravated when compared to the ideal minimum wage needed for a family of four, which in March 2020 should be R$4,483.20 (i.e. US$830.22 – 4 times the current value) (DIEESE, 2020). In February 2020, CadÚNICO had more than 28.1 million registered families, totaling almost 75 million people. Of these, more than 16.2 million families had a per capita income of up to R$178.00 (US $32.96), and 5.8 million were in the range between R$178.00 and half a minimum wage (US$96.76) (Ministério da Cidadania, 2020d).

On April 2nd, 2020,^8^ Emergency Aid was sanctioned (Brasil, 2020d), to mitigate the effects of the reduction of economic activity on the income of informal workers and those with low social protection during the pandemic period. As it is social workers, that form the front line of managing the benefits policy, it is important to reflect that these professionals tend to better understand the demands of the poor population. One of these benefits is Emergency Assistance.

## The emergency aid and the work of social workers

Emergency Assistance in the amount of R$600.00 (approximately US$123) was instituted initially for three months for a maximum of two people per family.^9^ This amount can be doubled in the case of women heading up a single parent family. The Ministry of Citizenship is responsible for managing this benefit, which was made available on April 17th, 2020, 10 days after its promulgation (Ministério da Cidadania, 2020c).

To access this emergency assistance, CadÚnico provided the infrastructure for payment. All families registered with CadÚnico by March 20th, 2020, who fulfilled the assistance conditions, automatically became beneficiaries - approximately 51.4 million Brazilians (70% of those registered) (Ministério da Cidadania, 2020b). To register those people not enrolled in CadÚnico by that date, a digital platform (cell-phone application or website) from the Caixa Econômica Federal bank was adopted. However, accessing emergency assistance has presented challenges for both the claimants and SUAS, either due to difficulties in registering (as not all possible beneficiaries have internet access), a lack of information on the assistance criteria^10^ and the physical overcrowding of the banks and lottery agencies that make the payment (which then violates social distancing guidelines). This emergency measure did not take into consideration the structural condition of the 5,570 Brazilian municipalities, of which 89.1% have fragile institutional management structures, rudimentary technical and administrative routines and reduced and/or poorly qualified human resources (Raichelis, 2010). In addition, the consequences of the pandemic have increased the demand for other benefits, such as funeral assistance (Brasil, 2020f) and food supplies, intensifying the work demands on the teams that administer the Social Assistance Policy.

Currently, of the professionals working at SUAS, around one in five are social workers, with a considerable portion of them having precarious and/or temporary employment relationships.^11^ Largely, it is women, affected by gender inequalities, who, in the current context of the health crisis, are more likely to be inundated and overloaded with paid and unpaid work (domestic work and work as caregivers).

The social assistance services reference teams, which include social workers, have experienced a growth in demand, including: for the supply of material provisions, access to income, guidance on social distance measures, services to those at the highest risk of contagion, as in the case of the elderly (especially those in situations of neglect), homeless people, refugees, migrants, those at risk of domestic violence, etc. All of the demands are taking place in a context of requiring immediate assistance. The work of social workers requires an understanding of the socioeconomic and cultural determinants of social inequalities (CFESS, 2011), in this context, understanding the material demands required in the daily struggle of the working class to survive in an extremely unequal country.^12^

Although Social Assistance Policy is aimed at being an essential area for supporting people who are suffering the consequences of the pandemic, this has not resulted in greater recognition, appreciation and protection for workers administering the assistance, in contrast to health professionals for example. Added to this is the absence of personal protective equipment including masks, gloves, alcohol gel, for these workers along with the difficulties in including them as a priority for vaccination against H1N1 and COVID-19 rapid testing.

Brazilian social workers have facined work related and ethical challenges^13^ with employment rights and regulations for the support of professionals, such as, decent working conditions in order to guarantee the quality of professional practice (Brasil, 1993; CFESS, 1993). According to the values and role of the profession, it is the duty of social workers to participate in relief programmes for the population in situations of crisis, attending to and defending the needs and interests of the population (Brasil, 1993; CFESS, 1993).

Social workers, as public servants (78,16%), are involved in the delivery of assistance benefits (Raichelis, 2010). It is worth noting that there are difficulties for both those who deliver services and those who receive the services in the achievement of their respective guaranteed rights. Violations can be observed arising from the difficulties from population for the accessing the support include benefit: the assessments made by social workers; due to the limits of remote working; in the precarious working conditions in place long before the pandemic (but which are now getting worse); the reduced number of professionals available for delivering a satisfactory service; misunderstanding on the part of managers about professional’s attributes and competences; in delays in granting aid; in the reduced offer of other available benefits (such as food) among others. Faced with such challenges, the Federal Council for Social Work has published orientation and provided some guidance measures to these workers, so that they can claim their rights in the context of the health crisis and its repercussions (CFESS, 2020a, 2020b).

## Final reflections

These reflections show us that about 121 million Brazilians, around 57% of the country's population (estimated at approximately 211 million people) (IBGE, 2020b), are currently in a situation of poverty. When we add the millions of people enrolled on CadÚnico, those who have already applied for emergency aid and those judged eligible to receive aid, we have a population invisible to the government and its public policies. This is a consequence not only of the limited coverage and quality of services offered by social policies, the impact of the crisis for capital and its consequences for the country, but also the limitations imposed on the reproduction of the workforce by increasingly precarious jobs with limited employment rights.

We understand that, although it is not the responsibility of social workers to resolve the social contradictions posed by capitalism, the work of this profession is extremely important, as it is called upon to address its impact and effects. When developing and implementing social programmes and projects that enable access to social rights and the granting of social benefits, Brazilian social workers collaborate for the immediate reproduction of the workforce, especially of the most impoverished population. But this is a challenge that not only confronts social workers and civil servants who work in public policies, but all workers. It concerns the fight for the defence and expansion of universal, public, free and high-quality policies that meet the demands of the working class.

In the case of Brazil, the potential increase in poverty and inequality during and after the pandemic stresses the urgent need to implement a broad and comprehensive social protection system which is sensitive to the different realities of the country and, crucially, as guided by the Constitution, guarantees financing for its full implementation. To this end, it is essential for the profession and wider society to vigorously debate the importance and availability of Emergency Aid, to ensure that social protection policies and interventions are firmly within the scope of social security policies. Furthermore, it is critical to reconnect and enhance the struggle of the working classe for social rights, by employing in the broadest sense all available professional strategies. In this context, the work and experience of social workers is important to understand the limits of the effectiveness of these benefits, societies rights and the impacts on people and society. Following the immediacy of the pandemic we will need to review not only how Covid-19 has highlighted existing inequality and fractures in Brazilian society but also the standards for sociability and social protection.

## Notes

1 In accordance with Decree Nº 10.316 of 7th April 2020 (Brasil, 2020b) and Law Nº 13.982 of 2nd April 2020 (Brasil, 2020d)

2 At the time this essay (20th October 2020) was completed the official figures were 5,250,727 cases and 154,176 deaths (Ministerio da Saúde, 2020).

3 Benefits under Social programmes are issued via this registration; it is one of the instruments that informs the National Information System of the Unified System for Social Assistance (SUAS network), overwhelmingly used by Social Workers. This Register includes families with monthly income from half the minimum salary per person, up to 3 times the minimum salary (approximately US$580) (Brasil, 2011).

4 Yazbek (2010) defines poverty as being a multidimensional category, which is expressed not only as a lack of material good, but also by the lack of rights, opportunities, information, possibilities and future aspirations.

5 Article 6 – “The social rights are education, health, food, work, housing, transportation, leisure, security, social security, maternity and child protection, assistance to the destitute […]” (Brasil, 1988).

6 According to microdata from the Cadastro Geral de Empregados e Desempregados (CAGED) until December 2019 (Secretaria de Trabalho and Ministério da Economia, 2020).

7 Constitutional Amendment Nº. 95 (2016) fixed the total spending on the public service offer at the 2017 rate for the next 20 years (Brasil, 2016). This Constitutional Amendment on Spending Ceilings instituted a New Fiscal Regime within Budget Planning and Social Security budgets, limiting spending for the next 20 years (Brasil, 2016)

8 On the same date, Provisional Measure N^o^. 937 made available an credit line worth R$98.2 Billion to finance, among other things, the Emergency Aid Programme (Brasil, 2020e).

9 Actually the Emergency Assistance was extended until December 2020.

10 The SUAS structure was not involved in the registration of new beneficiaries.

11 According to the SUAS census of 2018 and 2019 (Ministerio da Cidadania, 2020a).

12 Baqui et al. (2020: e1018) demonstrated that in Brazil are two distinct but associated effects with COVID-19 deaths: “increased mortality in the north region (regional effect) and in the *Pardo* and Black populations (ethnicity effect)”. Both effects are associated with poverty.

13 Between March and September 41 social workers died for COVID-19 (CFESS, 2020c). In total, we have in Brazil approximately 190,000 social workers registered (CFESS, 2020a).
